# The need for a coordinated international pandemic response

**DOI:** 10.2471/BLT.20.020620

**Published:** 2020-06-01

## Abstract

The COVID-19 pandemic has drawn attention to the international agreement governing responses to public health emergencies, with some experts calling for its revision. Lynn Eaton and Gary Humphreys report.

Dr Seif Al-Abri, Director General of Disease Surveillance and Control at the Ministry of Health in Oman, learned of his government’s decision to suspend flights to and from China after leaving a meeting of the National Committee for Civil Aviation Security and Facilitation.

The 3 February meeting had been convened to consider the country’s COVID-19 pandemic response. Al-Abri had told the committee that the World Health Organization (WHO) did not recommend travel or trade restrictions, a recommendation that had been repeated on 30 January by WHO Director-General Tedros Adhanom Ghebreyesus when he declared the COVID-19 outbreak to be a Public Health Emergency of International Concern (PHEIC).

“I left the meeting after giving my advice, and a half hour later I was told we would be suspending flights to and from China,” Al-Abri says. “I subsequently told my WHO contacts, I’m sorry, but at the end of the day, the decision about what to do in the face of a pandemic is a question for national governments.”

That question has been faced by governments worldwide since the COVID-19 outbreak began and most have decided to introduce travel and trade restrictions.

WHO’s consistent recommendation is that countries avoid trade and travel restrictions where possible. This recommendation is based on evidence suggesting that such restrictions may slow the spread of disease, but do not prevent it, while impacting the global economy and people’s wellbeing.

Travel and trade restrictions also disrupt the supply chains vital to pandemic response efforts.

“Travel and trade restrictions imposed during the COVID-19 pandemic, have had a significant impact on the delivery of equipment needed for personal protection of health workers, ventilators and other intensive care unit equipment,” says Dr David Heymann, professor of infectious disease epidemiology at the London School of Hygiene & Tropical Medicine. “They have also hampered the transfer of humanitarian workers including experts needed to give technical support for outbreak control including testing and contact tracing.”

There is also a concern that travel and trade restrictions – or rather the fear that they might be imposed – act as an incentive for countries to withhold information regarding outbreaks, especially in their earliest stages, when timely reporting is so important to effective outbreak responses.

Avoiding unnecessary interference with international traffic and trade is a core aim of the *International Health Regulations* (IHR), an international legal agreement on infectious disease response and other health emergencies, which was established in 1969 and was revised by the Member States of WHO in 2005.

“A key area of concern is any restriction on the movement of medical supplies, or other humanitarian aid.”

David Heymann.

The IHR call for countries to develop public health capacity to cope with disease outbreaks and other events, share information in a timely manner about outbreaks and implement response measures that are commensurate with the risk posed, supported by science, and anchored in human rights.

According to Gian Luca Burci, adjunct professor of international law at Geneva’s Graduate Institute of International and Development Studies, and former WHO legal counsel, the IHR originate from sanitary treaties drawn up in response to disease outbreaks in the 19th century. Burci explains that the IHR do not technically constitute a treaty, but are closer to a binding resolution by the United Nations Security Council.

The IHR are based on the understanding that diseases do not respect borders, that outbreaks in one country can easily spill into others and that evidence-based, international responses are required to deal with them.

In other words, the IHR are founded on the understanding that decisions about what to do in the face of pandemics, while ultimately “a question for national governments,” are best taken by national governments working together, sharing information and lessons learned, and relying on the best scientific evidence available.

These ideas were well understood by the states who committed to supporting the IHR in 2005. So, the question arises: why, in the unfolding COVID-19 pandemic, have so many states not adhered to core IHR precepts?

In the past, answers to this question have sometimes touched on the issue of enforceability. In 2011, for example, the IHR review committee, considering the functioning of the IHR in relation to the H1N1 pandemic, stated that “the most important structural shortcoming of the IHR is the lack of enforceable sanctions.”

In 2014, the Ebola Interim Assessment Panel came to similar conclusions, stating that the global community did not ‘take seriously’ its IHR obligations, and recommending that the IHR Review Committee ‘examine options for sanctions’ against such measures.

For Dr David Nabarro, Special Adviser to the United Nations Secretary-General on the 2030 Agenda for Sustainable Development and Climate Change, and WHO veteran of numerous infectious disease outbreaks, discussion about introducing enforceable sanctions into the IHR tends to overlook an important fact.

“Whenever somebody says the IHR or WHO should have more teeth, they need to consider the reality that the multi-national system is the property of the Member States and can only work within the remit given it by those states,” he says.

For Burci, the lack of enforceable sanctions does not make the IHR any less binding. “Other forms of accountability, such as peer pressure, naming and shaming mechanisms, or joint processes to assess compliance, can provide incentives or deterrents and improve compliance," he says.

Nabarro takes a similar view, noting that, in the international sphere, there are many agreements that have neither consequences nor enforcement mechanisms. “This does not mean that they do not have legal weight,” he says, citing as examples the WHO Framework Convention on Tobacco Control and the WHO Constitution.

Nabarro also points out that, while there are many examples of international law with “teeth”, including the World Trade Organization founding documents, which provide for contest and sanctions if a country is found to be in violation, it has not always been easy to get countries to sign up to them.

“The reality [is] that the multi-national system is the property of the Member States.”

David Nabarro.

“Agreeing to consequences is tantamount to ceding sovereignty to an external entity, which is quite antithetical to the notion of statehood under which the world is still operating,” Nabarro says.

That notion of statehood informed the 2005 revision and drafting of the IHR, article 4.3 of which states that nations have, “the sovereign right to legislate and to implement legislation in pursuance of their health policies.” It is that right that governments are exercising in the COVID-19 pandemic.

Nabarro believes that it is also important to recognize how much states are in compliance with the IHR, and how much progress has been made, notably in outbreak reporting, and data sharing.

Broadly in agreement with this assessment, Burci nevertheless believes the IHR would benefit from revision, notably with regard to the alert mechanism currently employed. “At present the IHR are binary – with outbreaks being a matter of international concern or not. There is no system of gradation,” he says.

Burci argues that this can result in poorly-calibrated, knee-jerk responses, often exacerbated by the media frenzy that typically follows the declaration of a PHEIC. Burci believes that there needs to be a way to reflect the evolution of an outbreak, possibly using the kind of colour alerts employed with other threat assessment systems.

Heymann also believes the IHR could be improved. “A key area of concern is any restriction on the movement of medical supplies, or other humanitarian aid,” he says. “There should also be some way of addressing non-governmental actors, including private corporations, such as airlines, which can impose de facto travel restrictions for their own reasons.”

The IHR Emergency Committee again convened on 30 April 2020, and made several recommendations to WHO, including continuing to work with countries and partners to enable essential travel needed for pandemic response, humanitarian relief, repatriation, and cargo operations.

The committee also asked WHO to update recommendations on appropriate travel measures and analyse their effects on international transmission of COVID-19, with consideration of the balance between benefits and unintended consequences.

Whether or not now is the time to open discussions on possible changes to the IHR is a matter for debate.

Heymann believes that there is a case for doing so, pointing out that the revision of the IHR in 2005 picked up momentum during the 2003 World Health Assembly. “That was during the height of the SARS outbreak,” he says.

Nabarro takes a different view. “If we are going to look at the IHR, I think we should do it later, when we – Member States, communities, and WHO itself – can spare the resources and expertise that are currently focused, entirely appropriately, on responding to COVID-19,” he says, adding that now is the time for leadership, at the country, regional and global level. “That means leadership that takes the full measure of the complex and evolving science about Covid-19 and implements robust, evidence-based policy.”

**Figure:**
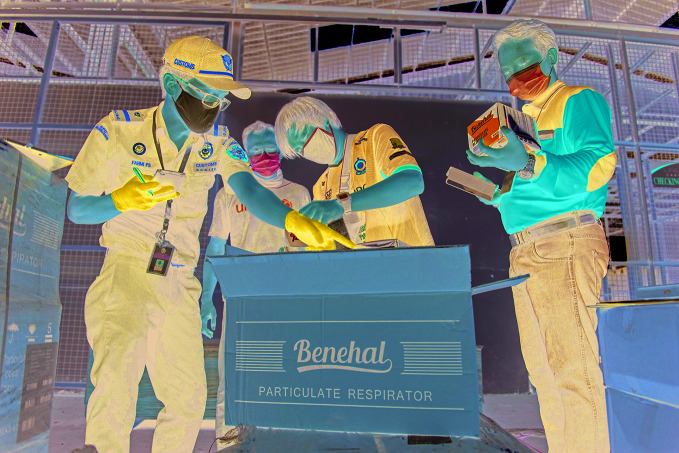
Customs officers inspect international aid goods at Soekarno Hatta Airport in Indonesia

UNICEF/Arimacs Wilander

**Figure:**
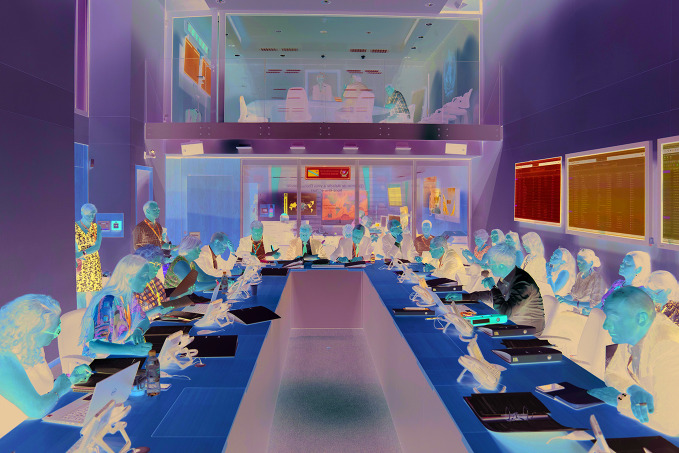
WHO staff facilitate a virtual meeting of the IHR emergency committee in the presence of its chair, Dr Robert Steffen, sitting to the right of WHO Director-General, in July 2019.

WHO/Christopher Black

